# UL11 Protein Is a Key Participant of the Duck Plague Virus in Its Life Cycle

**DOI:** 10.3389/fmicb.2021.792361

**Published:** 2022-01-04

**Authors:** Linjiang Yang, Mingshu Wang, Anchun Cheng, Qiao Yang, Ying Wu, Juan Huang, Bin Tian, Renyong Jia, Mafeng Liu, Dekang Zhu, Shun Chen, Xinxin Zhao, Shaqiu Zhang, Xumin Ou, Sai Mao, Qun Gao, Di Sun, Yanlin Yu, Ling Zhang

**Affiliations:** ^1^Research Center of Avian Diseases, College of Veterinary Medicine, Sichuan Agricultural University, Chengdu, China; ^2^Institute of Preventive Veterinary Medicine, Sichuan Agricultural University, Chengdu, China; ^3^Key Laboratory of Animal Disease and Human Health of Sichuan Province, Chengdu, China

**Keywords:** Duck plague virus, lipid raft, replication, prohibition, UL11

## Abstract

Tegument protein UL11 plays a critical role in the life cycle of herpesviruses. The UL11 protein of herpesviruses is important for viral particle entry, release, assembly, and secondary envelopment. Lipid raft is cholesterol-rich functional microdomains in cell membranes, which plays an important role in signal transduction and substance transport. Flotillin and prohibition, which are considered to be specific markers of lipid raft. However, little is known about the function of duck plague virus (DPV) UL11 in the life cycle of the viruses and the relationship between the lipid raft and UL11. In this study, an interference plasmid shRNA126 for UL11 was used. Results showed that UL11 is involved in the replication, cell to cell spread, viral particle assembly, and release processes. Furthermore, UL11 was verified that it could interact with the lipid raft through sucrose density gradient centrifugation and that function correlates with the second glycine of the UL11. When the lipid raft was depleted using the methyl-β-cyclodextrin, the release of the DPV was decreased. Moreover, UL11 can decrease several relative viral genes mRNA levels by qRT-PCR and Western blot test. Altogether, these results highlight an important role for UL11 protein in the viral replication cycle.

## Introduction

Herpesviruses are enveloped by double-stranded DNA viruses, which could cause medically and economically important diseases in humans and animals. Herpesviruses can be divided into three subfamilies, including *Alphaherpesvirinae, Betaherpesvirinae*, and *Gammaherpesvirinae*. Herpesviruses mainly affect the skin and mucosa and seriously harm humans and other animals’ health (Yuan et al., 2007; Qi et al., 2008, 2009). In *Alphaherpesvirinae*, which includes herpes simplex virus 1 and 2 (HSV-1/2), varicella-zoster virus (VSV), pseudorabies virus (PRV), Marek’s disease virus (MDV), equine herpesvirus (EHV), and duck plague virus (DPV). Duck plague, also known as duck viral enteritis (DVE), is an acute, febrile, and septic infectious disease in ducks, geese, and other *Anseriformes* (Cheng, 2015). The DPV is epidemic in all kinds of internal organs, blood, secretions, and excreta in infected ducks, and the highest amount was found in the liver, lung, and brain. Under natural conditions, the disease mainly occurs in ducks and is susceptible to ducks of different ages, genders, and breeds. Muscovy duck and mallard duck are the most susceptible, followed by Peking duck. The incubation period of natural infection is usually 2–4 days. Incidence of the disease is less in ducklings within 30 days old (Dhama et al., 2017).

Herpesviruses undergo two forms of replication, lytic replication, and latent infection. In the lytic replication cycle, before the virus enters a cell, it first attaches to a cell and invades into the cell, then the viral DNA begins to replicate after the capsid DNA is released into the nucleus. Subsequently, after assembly and genome packaging, the capsid leaves the nucleus (You et al., 2017). The viral particles undergo primary envelopment and de-envelopment at the nuclear envelope, with tegumentation and secondary envelopment occurring in the cytoplasm. Finally, the virions leave the host by exocytosis (Mettenleiter et al., 2009; Johnson and Baines, 2011; Zeev-Ben-Mordehai et al., 2014). In the herpes virus’s life cycle, many proteins participate in it, especially the tegument proteins. As we all know, herpesviruses are composed of three kinds of proteins; among them, tegument proteins play key roles in viral entry, secondary envelopment, viral capsid nuclear transportation during infection, and immune evasion (Owen et al., 2015; Yang et al., 2019). Although the absence of UL11 in HSV and PRV revealed only moderate defects in viral replication, the human cytomegalovirus (HCMV, or HHV-5) UL11 homolog UL99 is essential for viral replication (Britt et al., 2004). The function of UL11 includes affecting the primary and secondary envelopment of viral particles and the release of viral particles. Recent studies have found that UL11 can bind to rRNA to play a certain function (Claire et al., 2020). Nonetheless, there are few studies for DPV UL11.

Lipid rafts are composed of membrane microdomains that are riched in cholesterol and glycosphingolipids. It is also called DRMs (detergent-resistant membranes). These microdomains are specialized and heterogeneous cellular membrane subdomains defined by their resistance to solubilization with cold non-ionic detergents (Brown and Rose, 1992; Koshizuka et al., 2007). Lipid raft contained specific proteins, including stomatin, prohibitin, flotillin, and HflK/C (SPFH) domain proteins (alternatively termed stomatin-domain proteins) being found in lipid rafts in various cellular membranes (Browman et al., 2007). Lipid raft is located mainly on the plasma membrane, but raft assembly often occurs in the Golgi apparatus (Simons and Ikonen, 1997). Proteins with raft structures are key mediators of many biological events, such as trafficking and signal-transduction pathways. Moreover, lipid rafts affect several viruses’ replication cycles, such as platforms for virus entry, assembly, and budding (Briggs et al., 2003; Chazal and Gerlier, 2003; Lee et al., 2003; Manes et al., 2003).

We verified the function of UL11 by downregulating the UL11 expression by shRNA. By knocking down the expression of UL11 protein, we carried out a viral adsorption experiment, invasion experiment, replication experiment, assembly, release experiment, and cell to cell transmission experiment to verify the specific function of UL11 protein in the virus life cycle. Moreover, we identified that UL11 interacts with the prohibition and flotillin, providing a base for future studies about the correlation between the UL11 and the lipid raft.

## Materials and Methods

### Cells and Viruses

Duck embryo fibroblasts (DEF) cells were cultured in the Dulbecco’s modified Eagle’s minimum essential medium (DMEM) supplemented with 10% newborn bovine serum calf serum (NBS) at 37°C. Human embryonic kidney (HEK) 293T cells were maintained in 1,640 (Gibco Thermo Fisher Scientific, United States) supplemented with 10% fetal bovine serum (Thermo Fisher Scientific, United States), 100 U/mL penicillin, and 100 μg/mL streptomycin at 37°C in a 5% CO_2_ atmosphere. DPV in this study was the stock in our lab.

### Plasmids

The shRNA vectors are pGPU6-GFP and pGPU6. The interference plasmids shRNA-37 (CTGCAGAAGAAA CATATTAAC), shRNA-126 (CCGACAATGACAACTTTGA GA), shRNA218 (GATAGGCGTTCTTGTTATAGA), and shRNAneo (CCGACAATG ACAACTTTGAGA) were constructed by Gene Pharma (Gene Pharma, CHN). The pCAGGS-UL11-3 × Flag, pCAGGS-UL11G2A-3 × Flag, pEGFP-N1-UL11 plasmids were used in the homologous recombination method for cloning. pCAGGS-UL11G2A-3 × Flag (were site-directed mutated by point mutation kit (Transgen, CHN) (Yang et al., 2021).

### Antibody

The rabbit antibodies of *gB*, *UL21*, gE, and mouse antibodies of Us3, UL16, ICP27, ICP22 were provided by our lab (Yang et al., 2021). Anti-Flag monoclonal antibody was bought at the MBL with a dilution of 1:3,000 (MBL, JPN). Anti-flotillin monoclonal antibody was purchased from Abcam with a dilution of 1:3,000 (Abcam, United States). The anti-GFP monoclonal antibody was purchased from Transgen with a dilution at 1:1,000 (Transgen, CHN), and the anti-prohibition antibody was purchased from Thermo with a dilution at 1:1,000 (Thermo Fisher Scientific, United States).

### Viral Growth Curves

DEF cells were grown in 12-well plates, and the shRNA interference plasmids were transfected into the DEF cells. The cells were infected with 0.01 MOI DPV virus. After 1 h of infection, cells were washed with PBS three times and grown in a culture medium. At various time points post-infection, samples were harvested by freezing at −70°C.

### Viral Adsorption and Invasion

When the DEF cells grew to 90% for the viral adsorption assay, the shRNA interference plasmids were transfected into the DEF cells. One hour before infection, the DEF cells were incubated at 4°C for 1 h. Then infected with 1 MOI DPV virus, and immediately incubated at 4°C for 2 h. The cells were washed with pre-cooled PBS 5 times. The cell samples were collected for virus copy number detection. When the DEF cells grew to 90% for the invasion test, the shRNA interfere plasmids were transfected into the DEF cells. One hour before infection, the DEF cells were incubated at 4°C for 1 h. Then inoculated with 1 MOI DPV virus and immediately incubated at 4°C for 2 h. The cells were washed with pre-cooled PBS 5 times, changed to a cell maintaining culture solution. After incubation for 3 h at 37°C, the cell samples were collected for virus copy number detection.

### Plaque Assay

When the DEF cells grew to 90% for the plaque assays, the shRNA interfere plasmids were transfected into the DEF cells. The DPV was diluted to four gradients and infected with DEF cells, respectively. The cells were incubated at 37°C for 2 h. Then the supernatant was discarded and replaced with a 1% methylcellulose medium. The cells were cultured in a 37°C 5% CO_2_ incubator for 4–6 days. Then, the methylcellulose was discarded, and 4% paraformaldehyde was added to each well to fix at room temperature for 15 min; then, the cells were washed twice with sterilized PBS, stained with 0.5% crystal violet for 5 min, and washed with tap water to remove the staining solution, and the size of plaque was captured. The diameter of 25 plaques was measured by Photoshop software (Albecka et al., 2017).

### Plaque Morphology Analysis

When the DEF cells grew to 90%, the shRNA interfere plasmids were transfected into the DEF cells. Then, cells were infected with 0.001 MOI DPV. After incubation at 37°C for 2 h, 1% methylcellulose (Solarbio, Beijing, China) was added to cover the cells. At 60 hpi, the cells were observed under a fluorescence microscope (Nikon TI-SR, Japan). Twenty selected green fluorescent plaques were photographed for each virus, and the average plaque size was measured using Image J.

### Subcellular Fractionation

HEK 293T cells were transfected with pCAGGS-UL11-3 × Flag or pCAGGS-G2AUL11-3 × Flag. After 24 h post-transfection, cells were washed twice with ice-cold TNE buffer (25 mM Tris-HCl [pH 7.4], 150 mM NaCl, 5 mM EDTA) and lysed with TNE buffer containing 1% Triton X-100 and a protease inhibitor cocktail (Beyotime, CHN). Cell lysates were incubated on ice for 20 min and then were broken by passage through freeze-thaw. After centrifugation at 800 g for 5 min to remove the nuclei, the cell lysates were adjusted to 40% (w/w) sucrose, overlaid with 30 and 5% sucrose in TNE buffer centrifuged at 200,000 g for 16 h. The gradient was fractionated from the top (Fraction No. 1) to bottom (Fraction No. 9) and resuspended in sodium dodecyl sulfate-polyacrylamide gel (Koshizuka et al., 2007).

### Immunofluorescence Analysis

Cells grown on coverslips were washed three times with PBS and fixed overnight with 4% paraformaldehyde in PBS at 4°C. For the indirect immunofluorescence analysis (IFA), the fixed cells were permeabilized with 1% Triton X-100 in PBS for 20 min at room temperature and then incubated with blocking buffer (3% bovine serum albumin in PBS) for 1 h at room temperature. Then, the cells were incubated with primary antibodies and Alexa Fluor-conjugated secondary antibodies (at a dilution of 1:1,000) in a blocking buffer for 60 min at room temperature. The samples were examined under a Nikon H550L fluorescence microscope (Yang et al., 2020).

### Cholesterol Depletion Assay

Transiently expressing DEF cells were incubated at room temperature in either serum-free DMEM or serum-free DMEM supplemented with 7 mM methyl-β-cyclodextrin (MβCD) (Sigma, United States) to deplete cholesterol. After 30 min of incubation, cells were infected with DPV at an MOI of 1 and incubated for an additional 1 h at 37°C. Afterward, cells were washed with PBS and cultured with 2% NBS DMEM (Dietz et al., 2017).

### Electron Microscopy

DEF cells in a 60 mm plate were first transfected with shRNAs, then after 12 h infected with 2 MOI DPV. Furthermore, harvested the cells at 14 h post-infection. After centrifugation at 1,500 rpm for 10 min, the supernatant was discarded. Slowly add 0.5% glutaraldehyde (diluted with PBS 1:6) along the tube wall with a pipette, and let it stay at 4°C for 10 min. Centrifugation was performed at 10,000–13,000 rpm for 10–15 min, and the supernatant was discarded. In addition, slowly added 3% glutaraldehyde fixed solution along the tube wall in the pipette. Lastly, we sent the samples to the LILAI Medical Experimental Center for electron microscopy (LILAI Biotechnology, CHN). The enveloped and membrane attached percentage result is based on the proportion of enveloped virus particles in the total number of enveloped and non-enveloped virus particles.

### Cell Viability Detection

The 96 well plate monolayer cells were grown for 24 h and then incubated with different concentrations of drugs for 30 min at room temperature, then washed with PBS for 3 times, and incubated with cell activity detection solution CCK-8 (Boster Biological Technology Co., Ltd., United States) at 37°C for 1–2 h, eventually detected with 450 nm enzyme-labeled instrument (Bio-rad, United States).

### Protein Mass Spectrometry

SDS-PAGE was used to separate UL11 IP protein. The products were sent to the PTM BIO (JINGJIE, CHN) stained 200 with Coomassie brilliant blue (Bio-Rad, United States) and then sent to PTM Bio Company (PTM Bio, CHN) for liquid chromatography-tandem mass 1.3. spectrometry Proteome Discoverer Tandem mass (LC-MS/MS) analysis.

### Quantitative Reverse Transcription PCR

When the cells are grown for 24 h and then transfected with shRNAs. After transfection for 12 h, the cells were infected with the DPV. The total RNA was isolated from the DPV-infected DEFs, and reverse transcription was performed; an uninfected control was included. The primers were designed with Oligo 7 (Table 1). Quantitative reverse transcription PCR was performed in a 20-μL reaction volume containing 10 μL of SYBR Green mix (Takara, JPN), 1 μL of each primer, 1 μL of cDNA, and 7 μL of RNase-free water. Triplicate experiments were performed to analyze UL21, UL11, UL16, ICP4, ICP22, ICP27, US3, gE, gB, and β-actin gene expression, and the relative transcription levels were calculated using the 2-ΔCt method.

**TABLE 1:** Sequence and characteristics of qRT-PCR primers.

Primer	Primer sequence (5′–3′)	Gene	Product size (bp)
F	TACGCCAACACGGTGCTG	β-actin	178
R	GATTCATCATACTCCTGCTTGCT		
F	GCCCAGGAACACCAGTCT	DPV UL21	106
R	CAGTGCGTATTGCCGTCT		
F	ATGGGACAAGCTCAATCCTAC	DPV UL11	144
R	AAAGTTGTCATTGTCGGTTGG		
F	CTCGGGCATTGAACACACAA	DPV UL16	153
R	TTATTTCCACCATTGCCGCC		
F	GAACAACCGCCGAACAC	DPV ICP27	127
R	TCAAACATCCGCCTCAA		
F	CGTAGCGTCACATCAAGCAG	DPV ICP22	147
R	GCGTTTGGTCCCTATAACCTC		
F	CGTTCGCTCAGCTATACCCT	DPV ICP4	196
R	GGTCCGCTTATACTGAGTCCA		
F	AAAATAACATCGTGGGC	DPV US8	112
R	TTCGGTAGACTTTAGCATC		
F	GCACGGAAGAGGAATAATACACC	DPV US3	148
R	CGTTCCAACCCACCCATAGTC		
F	CTTACTCCCAGGGGTGAA	DPV UL27	205
R	ACTTTTTCCCATTTGACCTCG		

### Statistical Analysis

All statistical analyses were performed using GraphPad Prism 6.0 software (La Jolla, CA, United States). Unpaired *t*-test and multiple *t*-tests were used to determine statistical significance. All experiments were repeated at least three times individually. The data was expressed as the mean and standard error of the mean (SEM). Values of *P < 0.05 were considered statistically significant.

## Results

### Growth Analysis of UL11 Knockdown Viruses

**FIGURE 1:**
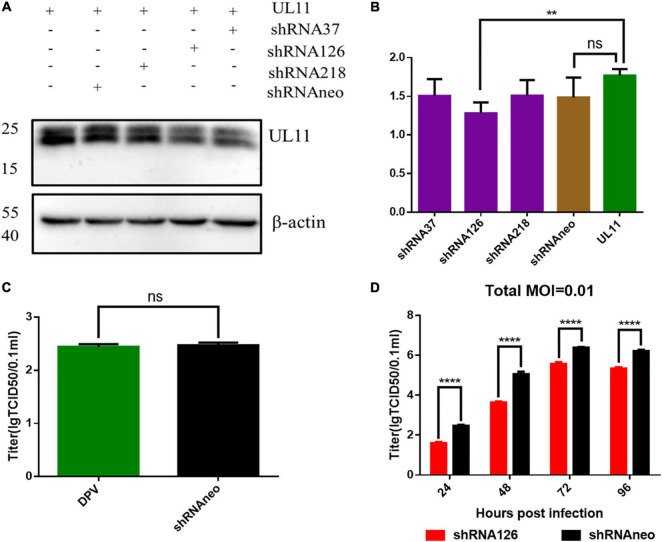
DPV growth curve analysis of interfering UL11 in DEF cells. **(A)** In the transfection condition, DEF cells were transfected UL11 with shRNA37, shRNA126, shRNA218, and shRNAneo, respectively. After 24 h, cells were collected to do Western blot analysis. **(B)** Quantification of the UL11 downregulation ratio. **P < 0.01; *^ns^P* > 0.05. **(C)** The effect of the shRNAneo on the replication of DPV. ^*ns*^*P* > 0.05. **(D)** Growth curve of the UL11 downregulated mutant. The DPV infection at 0.01 MOI. ****P < 0.0001.

Viral growth kinetics were detected to investigate whether UL11 protein plays a role in DPV replication. Three shRNA plasmids were transfected into the DEF cells to knock down UL11. First, shRNAs knockdown efficiency was determined through the Western blot. As a result, we found that in the transfection test, the shRNA126 can greatly decrease the expression level of the UL11 compared to the other two shRNAs (Figures 1A,B and Supplementary Figure 1). The DEF cells were transfected with the shRNAneo, 12 h before the cells were infected with DPV at the 0.01 MOI. Twenty-four hours after being infected with DPV, the DEF cells were harvested, and the DPV was titered by the TCID_50_ assay. Results showed that the production of DPV between the shRNAneo-treated group and the control group did not have statistical significance (Figure 1C), indicating the vector did not affect the replication of the DPV. Subsequently, a slight fluctuation in titers was observed from 24 to 96 hpi. Virus growth was further assessed in a multistep growth kinetics analysis by coculture of infected with non-infected DEF cells (Figure 1D). Virus yields of shRNA126 were decreased at least 10-fold compared to those of the shRNAneo group. Detailed mean value and SD were provided in Table 2. These results suggest that UL11 downregulation impaired DPV growth.

**TABLE 2:** The mean and SEM value of the growth curve.

Time	shRNA126			shRNAneo		
	Mean	SEM	N	Mean	SEM	N
24	1.603	0.032	3	2.470	0.030	3
36	3.635	0.032	3	5.067	0.067	3
48	5.579	0.048	3	6.392	0.018	3
96	5.344	0.035	3	6.222	0.034	3

### UL11 Is Not Required for the Adsorption and Invasion of Duck Plague Virus

**FIGURE 2:**
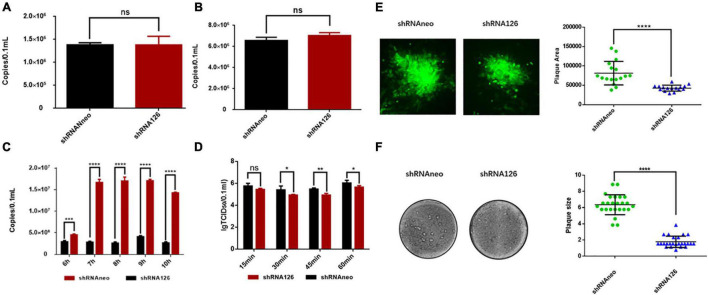
UL11 in the viral adsorption, invasion, replication, release processes, and cell to cell spread. **(A)** DEF cells were transfected with shRNA126 and shRNAneo, then inoculated with 1 MOI DPV to undergo the adsorption experiment. The cell samples were collected for virus copy number detection. ^*ns*^*P* > 0.05. **(B)** The shRNA126 and shRNAneo plasmids were transfected into the DEFs, then inoculated with 1 MOI DPV to detect invasion experiment. After incubation for 3 h, the cell samples were collected for virus copy number detection. ^*ns*^*P* > 0.05. **(C)** The shRNA interference plasmid was transfected into the DEFs, and then the cells were incubated with 1 MOI DPV to detect DPV replication. Cells were cultured at 37°C for 6, 7, 8, 9, and 10 h, the cells samples were collected for virus copy number detection. ***P < 0.001; ****P < 0.0001. **(D)** The shRNA interference plasmids were transfected into the DEFs, then the cells were incubated with 1 MOI DPV to detect DPV egress. The supernatant was collected and tested TCID_50_ in the culture for 15, 30, 45, and 60 min. ^*ns*^*P* > 0.05; *P < 0.05; **P < 0.01. **(E)** Green fluorescent plaques produced by the shRNA126 and shRNAneo. Statistical analysis of randomly selected viral green fluorescent plaques at the right. Plates were scanned, and plaque diameters were measured in Image J. ****P < 0.0001. **(F)** Crystal violet assay to test the cell to cell spread. Representative plaques are shown. 25 plaques per sample were measured to quantify the results at the right. Plates were scanned, and plaque diameters were measured in Photoshop. ****P < 0.0001.

Because the virus adsorption process is mostly related to the envelope proteins of the viruses, UL11 is a membrane-anchored protein and can interact with the envelope glycoprotein E (Han et al., 2011; Yang et al., 2020). The viral adsorption and invasion experiment were conducted to explore whether UL11 is involved in the virus adsorption process and investigate the function of the UL11 protein in the viral life cycle. Viral genome copies between the control and the interfering RNA groups were analyzed through the qPCR test. As shown in Figure 2A, there is no significant difference in the number of virus copies between the two groups. So, UL11 is not required for the adsorption process. Then, the viral invasion process was also be detected (Figure 2B), and the result was the same as the adsorption experiment. There was no difference between the control group and the interfering RNA group. All the results indicated that UL11 is not required for the DPV adsorption and invasion.

### UL11 Plays a Key Role in the Viral Replication and Release

A previous study verified that the US3 protein does not affect the production of viral genome copies (Deng et al., 2020). As a kind of herpesvirus tegument protein, whether DPV UL11 influences viral replication remains to be explored. As shown in Figure 2C, when the shRNA126 knocked down UL11, the viral copies number decreased greatly compared to the control group. There were significant differences between the two groups at 7, 8, 9, and 10 h after infection (*P* < 0.0001) (Figure 2C). Studies have reported that UL11 participated in the viral release process (Baines and Roizman, 1992). The viral release efficiency between the interfering and control groups was detected to investigate whether UL11 participates in the DPV release. Transfected DEF cells were infected with DPV and gathered the 15, 30, 45, 60 min samples after infection. The results showed that when UL11 decreased, the efficiency of the viral release decreased too (Figure 2D). However, the degree of decline is not as obvious as that of viral replication. Thus, the UL11 is important for the viral replication stage in the viral life cycle and slightly affects the release of mature virions.

### Effect of the Duck Plague Virus UL11 Protein on Viral Cell-to-Cell Spread

A previous study has reported that UL11 participated in the cell fusion process cooperating with the UL16, UL21, and gE (Han et al., 2012). Nevertheless, the cell transmission ability of the DPV UL11 is unclear. Therefore, a plaque morphology assay was conducted to explore the effect of the DPV UL11 protein on the cell to cell process between adjacent cells. To better assess green fluorescent plaque sizes, randomly selected green fluorescent plaques generated by the two strains were statistically analyzed. As a result, when UL11 was knocked down, the plaque size decreased significantly compared to the control group through the fluorescent plaque area detection. Representative plaques were shown, and the quantitative analysis results are shown below (Figure 2E). There was a significant difference in the size of plaque between the two. Since green fluorescent plaques did not mirror viral plaques, the crystal violet plaque assay was also conducted. By detecting the plaque size of the interfering group and the control group, we found that the interfering group had a smaller plaque size.

In contrast to the shRNA126, plaque sizes for shRNAneo are much bigger. Representative plaques were shown, and the quantitative analysis results are shown below (Figure 2F). There was a significant difference in the size of plaque between the two. Collectively, these data indicated that the DPV UL11 protein influenced the viral cell-to-cell spread process, coinciding with the significant reduction in viral titers caused by the interfered of UL11.

### UL11 Protein Participated in the Viral Secondary Envelopment Process

**FIGURE 3:**
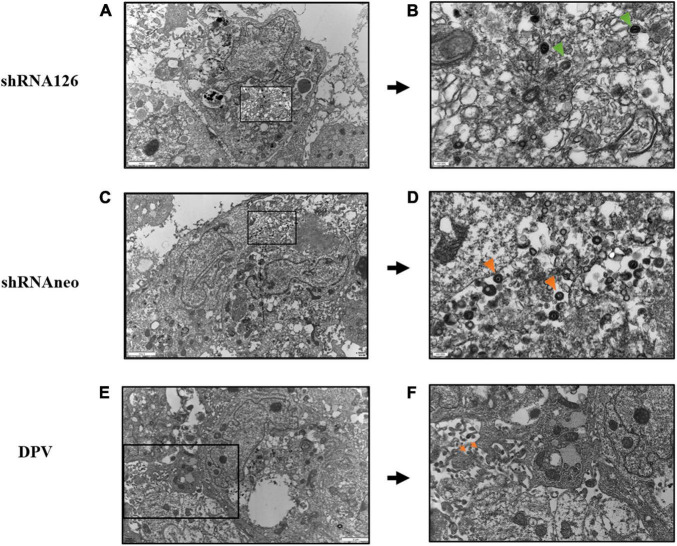
Electron microscopy analysis of DPV, shRNA126, and shRNAneo. **(A)** Electron micrographs of a DEF cell for the UL11 interfering group shRNA126. **(B)** Higher magnifications with close-ups of selected areas show incompletely enveloped particles for shRNA126. **(C)** Electron micrographs of a DEF cell for the shRNAneo group. **(D)** Higher magnifications with close-ups of selected areas show fully enveloped particles for shRNAneo. **(E)** Electron micrographs of a DEF cell for the DPV group. **(F)** Higher magnifications with close-ups of selected areas show fully enveloped particles for DPV. The orange triangle indicates the viral particles with complete envelopes, and the green triangle indicates the viral particles with membrane-attached.

The underlying cause of the growth and release impairment of a UL11 knockdown mutant is based on a defect in the secondary envelopment of virus particles. To visualize possible defects of UL11 on the DPV assembly process, we performed electron microscopy of virus-infected cells at 13 hpi. Enveloped virus particles are capsids with a tegument fully enclosed by a double membrane (envelope and vesicle membrane). Membrane-attached capsids are capsids at the process of secondary envelopment, which was defined as close contact to membranes that wrap around the capsid. Free capsids are those that have no contact with membranes (Dietz et al., 2017). As shown in Figure 3, cells were transfected with shRNA126 showed normal capsid morphogenesis in the nucleus with no obvious accumulation of capsids at the inner lamella of the nuclear membrane. However, the membrane attached virions accumulated in the DPV UL11 protein in the shRNA126 group compared with the shRNA neo and DPV-infected cells group. Also, the release of the virions was reduced. In shRNAneo and DPV, 68 and 69% enveloped virions were found, and only 23 and 19% were found membrane-attached, respectively (Table 3). However, virions in the shRNA126 group enveloped virions (34.3%) and membrane-attached (55.2%) were significantly different compared to the control, with a nearly 2-fold increase in membrane-attached virions (Table 3). The result indicated that the DPV UL11 protein affected virion secondary envelopment, impaired virions maturation.

**TABLE 3:** Ultrastructural quantification of secondary envelopment stages of DPV particles in vACs of infected DEF at 12 h post-infection.

Virus	No. of cells analyzed	No. of capsids per vAC	% Particles by type		
			Enveloped	Membrane attached	Naked
shRNA126	3	78	34.3	55.2	10.5
shRNAneo	3	76	68	23	9
DPV	3	65	69	19	12

### UL11 Knockdown Can Reduce the mRNA Level of Viral Genes

**FIGURE 4:**
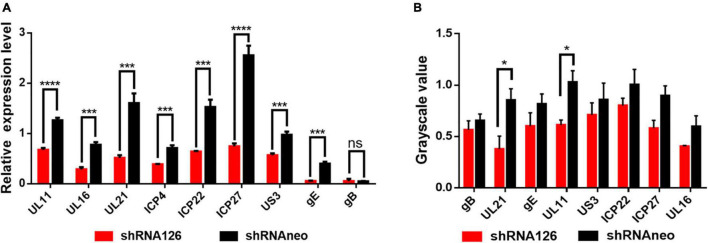
Effect on the other viral genes when downregulated the UL11. **(A)** mRNA level of UL11, UL16, UL21, gE, ICP22, ICP27, ICP4, US3, and gB. Transfected the shRNA126 into the DEF cells and then infected the cells with 1MOI DPV. After 24 h, the DEF cells were harvested and did the qRT-PCR. ^*ns*^*P* > 0.05; ***P < 0.001; ****P < 0.0001. **(B)** Protein expression levels of UL11, UL16, UL21, gE, ICP22, ICP27, US3, and gB when interfering with the UL11 gene. Transfected the shRNA126 into the DEF cells and then infected the cells with 1MOI DPV. After 24 h, the DEF cells were harvested and did the Western blot to undergo grayscale value. ^*ns*^*P* > 0.05; *P < 0.05.

With the above results, the reduced expression of the UL11 could affect the viral replication. The ICP22 (Fraser and Rice, 2007; Li et al., 2020), ICP27 (Tang et al., 2019), and ICP4 (Frasson et al., 2021) are immediate early genes, and UL11 (Yang et al., 2021), UL16 (Oshima et al., 1998; He et al., 2012), UL21 (Yang et al., 2020), gE (Po’ka et al., 2017) and gB (Ramachandran et al., 2010) are late genes, and the US3 (Labiuk et al., 2010) is an early gene. Nevertheless, how DPV UL11 takes part in the viral replication process, we still do not know. DEF cells were transfected with shRNA126 and then infected with 1 MOI DPV, and the transcription level of the UL11 was decreased a lot through the qRT-PCR. As shown in Figure 4A, the transcription level of the UL11, UL16, UL21, ICP4, ICP22, ICP27, US3, and gE decreased significantly, but gB did not change. The protein expression level of the proteins was also be analyzed through the Western blot (Supplementary Figure 2). Some genes’ mRNA levels may be decreased obviously, while the protein level has not changed much. Quantification of the protein level did not show significant change except UL11 and UL21 through grayscale value (Figure 4B). Thus, downregulation of UL11 can inhibit the UL16, UL21, ICP4, ICP22, ICP27, US3, and gE mRNA levels.

### UL11 Can Interact With the Lipid Raft and Depends on the Second Glycine

**FIGURE 5:**
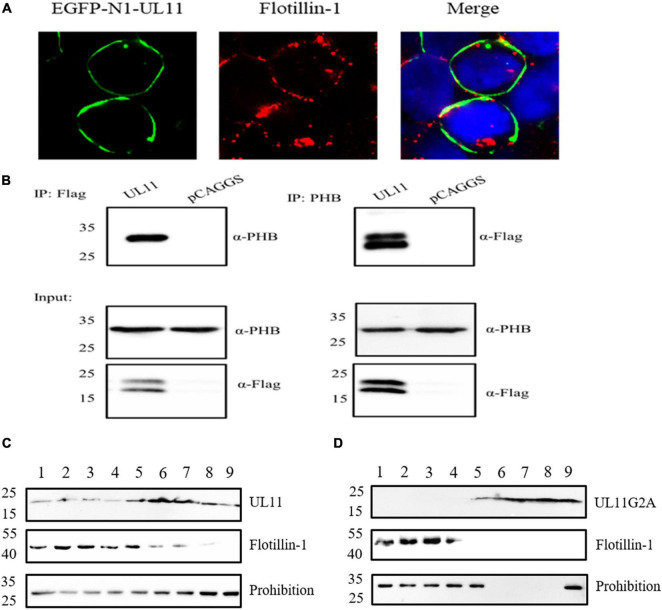
UL11 can interact with the lipid raft marker, flotillin-1, and prohibition. **(A)** DEF cells were transfected with EGFP-N1-UL11 for 24 h, then cells were fixed and did the Immunofluorescence analysis. The primary antibody is an anti-FLOT-1 monoclonal antibody, and the secondary antibody is Alexa Fluor 594 Goat anti-Rabbit IgG. The nuclei were stained with DAPI. **(B)** The co-immunoprecipitation between the UL11 and PHB. HEK 293T cells were transfected with UL11 and pCAGGS for 24 h. Then the samples were collected to do the co-immunoprecipitation. **(C)** As described in materials and methods, HEK 293T cells were transfected with UL11 and collected 48 h post-transfection. After removed of cell nuclei, cell lysates were subjected to sucrose-gradient centrifugation. Fractions were collected from the top of the tube. Proteins in each fraction were separated by SDS-PAGE and analyzed by Western blotting. **(D)** As described in Materials and methods, HEK 293T cells were transfected with UL11G2A and fractionated at 48 h post-transfection. After removed of cell nuclei, cell lysates were subjected to sucrose-gradient centrifugation. Fractions were collected from the top of the tube. Proteins in each fraction were separated by SDS-PAGE and analyzed by Western blotting.

UL11 is a membrane-anchored protein. To determine whether UL11 associates with lipid raft, protein mass spectrometry detection was conducted. Surprisingly, protein mass spectrometry identified that UL11 could interact with the lipid raft marker prohibition (Table 4). UL11 was transfected into HEK 393T cells and analyzed by confocal microscopy and sucrose gradient fractionation 48 h post-transfection. Wild type UL11 located at the plasma membrane by microscopy. Lipid raft was located at the plasma membrane through co-localization analysis and partially co-localized with the plasma membrane located wild-type UL11 (Figure 5A).

**TABLE 4:** Mass spectrometric results of lipid raft protein in DEF interact with UL11 protein.

Accession	Protein names	Gene names	MW [kDa]	Protein score	Sequence coverage (%)	#Unique Peptides	#Peptides	#PSMs
R0L8I2	Prohibition	Anapl-09413	30.12	35	3.64	1	1	1

The samples were detected by co-immunoprecipitation assay further to verify the relationship between the UL11 and lipid raft. The result showed that the UL11 pulled down the prohibition. Also, the prohibition could precipitate the UL11 (Figure 5B). Previously we determined that UL11G2A is important for the Golgi-apparatus and membrane localization (Yang et al., 2021). So the relationship between the G2A and lipid raft is worth exploring. DPV UL11 and UL11G2A were transfected into HEK 293T cells to undergo a sucrose gradient fractionations experiment. The solution was divided into 9 fractions. Furthermore, the samples were separated by SDS-PAGE and analyzed by Western blotting using anti-Flag and anti-FLOT1. As shown in Figures 5C,D, fractions contained FLOT-1 and PHB layers, UL11 also contained. In contrast, UL11G2A cannot be detected in the lipid raft fractions (Figures 5C,D). Hence, UL11 could interact with the lipid raft, which depends on the second glycine of the UL11.

### Effect of the Lipid Raft on Release and Spread

**FIGURE 6:**
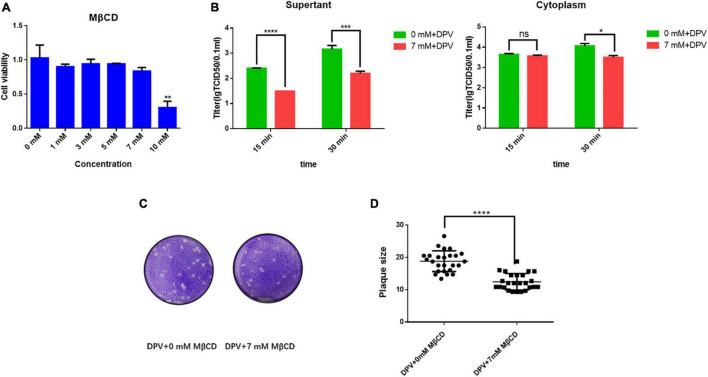
Lipid rafts take part in the DPV cell to cell spread and release processes. **(A)** Cell viability of adding MβCD. 1, 3, 5, 7, and 10 mM of MβCD was set, then the cell activity was detected. Moreover, 3 replicates were averaged. **P < 0.01. **(B)** MβCD was then diluted to a final concentration of 7 mM, a concentration that does not affect cells, and 1MOI DPV viruses were allowed to infect DEF cells. After incubation for 18 h at 37°C, the cell growth medium was replaced by a cell maintaining medium. The supernatant and cytoplasm were collected as samples, respectively, and the supernatant was put into 37°C for further culture. The supernatant and cytoplasm were collected and tested TCID_50_ separately in the culture for 15 and 30 min. The results presented are representative of three independent experiments. *^ns^P* > 0.05; *P < 0.05; ***P < 0.001; ****P < 0.0001. **(C)** MβCD was then diluted to a final concentration of 7 mM and different 10 x dilution gradient DPV were allowed to infect DEF cells for 5–6 days. After fixing, cells were stained with crystal violet. ****P < 0.0001. **(D)** Plates were scanned, and plaque diameters were measured in Adobe Photoshop. Data show plaque sizes on 25 plaques. ****P < 0.0001.

Lipid raft is a key component in viral infection. To determine the function of the lipid raft in the viral life cycle and the correlation with UL11, we did a drug experiment. Methyl-β-cyclodextrin (MβCD) is a well-known drug that depletes the lipid raft’s key composition cholesterol (Bender et al., 2003). As shown in Figure 6A, when treated DEF cells with 10 mM MβCD, the cell viability was impaired. So we chose 7 mM MβCD to treat the DEF cells. Because UL11 affected the viral cell to cell spread and release, whether lipid raft performed a similar function need to be determined. As Figure 6B, depleting the MβCD leads to the DPV release process being inhibited. So the lipid raft can take part in the viral life cycle of the release process. The lipid raft in the cell to cell spread process also is detected. As a result, the efficiency of the viral spread was decreased significantly, and the representative plaques were shown (Figure 6C) through crystal violet assay. Statistical analysis on the size of the plaques (Figure 6D) was analyzed. Similar to the viral release results, the viral cell to cell spread was also inhibited when depleted the cholesterol of the lipid raft consistent with the UL11. Thus, lipid raft is important for the viral release and cell transmission processes as the UL11.

In summary, in this study, we found that the DPV UL11 protein played a pivotal role in the viral life cycle by regulating viral cell-to-cell spread and secondary envelopment. UL11 can associate with the lipid raft, and the lipid raft is important for the DPV cell transmission and release. We hope that this study provides basic information about the DPV UL11 protein and a foundation for UL11 function research and DPV prevention and control.

## Discussion

Previous studies have reported that UL11 is non-essential for replication of HSV-1 (Baines and Roizman, 1992), PRV (Kopp et al., 2003), but the EHV-1 study showed that ORF51 encoding protein UL11 is an essential gene (Badr et al., 2018). In DPV, although transfection of cultured cells with UL11 deletion mutant BAC was successful, no CPE occurred, and no progeny viruses were recovered. These results suggested that the UL11 protein is essential for DPV replication in cultured cells, consistent with the EHV-1 (Badr et al., 2018). Our study cannot rescue the deletion mutant of the UL11, suggesting that UL11 is an essential gene, but we did not have the UL11 constitutively expressing cell line to determine.

The viral replication cycle includes adsorption, invasion, viral nucleic acid replication, cell to cell spread, assembly of nucleocapsids, maturation, and virus release (Owen et al., 2015). Virus adsorption is the first step of viruses infecting cells. For herpesviruses, in the early stage of infection, viral envelope glycoproteins adsorbed on the specific receptor on the cell surface, adsorbed virus particles swelled, the viral envelope fuse with the cell membrane, the plasma membrane at the adsorption site thickens, and the electron density increases. After the herpesvirus enters the cell and begins to shell, the virus particles disappear and enter the hidden period of virus infection. This covert phase is the most important stage in the process of viral proliferation. At this time, the genetic information of the virus is transmitted to the cells. Under the control of the virus genetic information, the cells synthesize various components of the herpesviruses and the required enzymes (Kobty, 2015). The newly synthesized viral components gradually mature and assemble into complete virus particles in infected cells. The maturation of herpesvirus particles needs to go through the process of the primary envelopment, de-envelopment, and secondary envelopment; that is, the virus undergo DNA replication in the nucleus, transcribes virus, assembles and encapsulates nucleocapsid, and then the nucleocapsid formed in the nucleus interacts with the inner layer of the nuclear envelope to obtain an outer membrane, which mediates the viruses to release from the nucleus in the way of budding. UL11 has multiple functions, which involve facilitating nucleocapsid envelopment and viral particles egress from the cells in HSV-1 (Baines and Roizman, 1992).

Also, HSV-1 gM, and UL11 are required for virus-induced cell fusion and efficient virus entry (Kim et al., 2013). In addition to this, EHV-1 UL11 participated in the cell to cell spread process and promoted cell to cell spread (Schimmer and Neubauer, 2003). In PRV, secondary envelopment can be affected by UL11 and gM. When constructing the UL11 and gM deletion strain, huge intracytoplasmic inclusions were observed. Plaque formation was virtually abolished by the simultaneous absence of UL11 and gM, and one-step growth was significantly reduced (Kopp et al., 2004). Thus, UL11 plays a key role in the viral replication cycle. Our study found that UL11 can participate in the replication, cell to cell spread, and egress processes.

Lipid raft is participated in the viral replication cycle, serving as a viral entry site, function for virion assembly, and acting as a scaffold for viral budding from infected cells (Lu et al., 2008; Rossman and Lamb, 2011). Also, in the Influenza virus, the hemagglutinin concentrates in lipid raft microdomains lead to efficient viral fusion (Takeda et al., 2003). Lipid rafts also harbor specific proteins, with stomatin, prohibitin, flotillin, and HflK/C (SPFH) domain proteins (alternatively termed stomatin-domain proteins) being found in lipid rafts in various cellular membranes (Yokoyama and Matsui, 2020). In HSV-1, gB may interact with a cellular molecule associated with lipid raft to mediate entry (Bender et al., 2003). HSV-2 UL56, a virion-host shutoff protein, VP16, and HSV-1 gH, can be associated with lipid raft in infected cells (Koshizuka et al., 2002). In PRV infected cells, gB, gC, gD, and gE can interact with the lipid raft (Favoreel et al., 2004). Our results demonstrate that UL11 is also associated with lipid raft microdomains. We used flotillin-1 as the lipid raft marker, suggesting that lipid raft may also play a role in the process of secondary envelopment. The relationship between the UL11 and other lipid raft-associated proteins will be further investigated in the future.

Not clear is why does UL11 traffic to the plasma membrane and DRMs? Some studies showed there are three possibilities. First, there might not be any function for UL11 on the plasma membrane. A recovery mechanism may be needed to solve how this protein returns to the budding site whenever it leaves the cell periphery. The problem with this model is that it does not explain why UL11 contains two recycling motifs (Loomis et al., 2001), only one of which is needed for packaging (Loomis et al., 2006). Because it is similar to the Nef protein (mentioned above) (Pereira and daSilva, 2016), a second model emerges is that UL11 travels to the plasma membrane and back to internal membranes for one or more specific purposes (e.g., to downregulate a host protein, to acquire a modification, or to bring another protein to the site of budding), which could be the sole purpose of UL11, or it could be formed as a bridge connecting capsids and membranes. The third is that UL11 travels to the plasma membrane to promote the cell-to-cell spread, either by enabling the egress of vesicle-enclosed virions to the cell surface or by promoting a direct interaction of the capsid (via UL16) with the plasma membrane (Chadha et al., 2012). The third one may be true in our study in DPV because DPV UL11 can interact with the prohibition. Our study showed that prohibitin contributes to the cell-to-cell transmission of HSV-1 via the MAPK/ERK signaling pathway (Watanabe et al., 2021), and UL11 can interact with gE, so maybe UL11 also take part in the cell to cell transmission process.

In summary, this study found that the DPV UL11 is a key participant in the viral life cycle by regulating the viral replication, viral assembly, cell to cell spread, and release processes. However, UL11 does not take part in the adsorption and invasion processes. Through an electron microscope experiment, we found that UL11 could affect the secondary envelopment of the DPV. Moreover, UL11 interacts with the lipid raft, which depends on the second glycine of the UL11. Lipid raft plays a key role in the viral cell to cell transmission and release processes that may lay the base for the relationship between UL11 and lipid raft in the cell transmission and release processes.

## Data Availability Statement

The original contributions presented in the study are included in the article/Supplementary Material, further inquiries can be directed to the corresponding author/s.

## Ethics Statement

The animal study was reviewed and approved by the Committee of experimental operational guidelines and animal welfare of Sichuan Agricultural University.

## Author Contributions

LY conceived, designed, and performed the experiments, analyzed the data, and drafted the manuscript. MW conceived and supervised the study. AC, QY, YW, JH, BT, RJ, ML, DZ, SC, XZ, SZ, XO, SM, QG, DS, YY, and LZ interpreted the data. All authors read and approved the final manuscript for publication.

## Conflict of Interest

The authors declare that the research was conducted in the absence of any commercial or financial relationships that could be construed as a potential conflict of interest.

## Publisher’s Note

All claims expressed in this article are solely those of the authors and do not necessarily represent those of their affiliated organizations, or those of the publisher, the editors and the reviewers. Any product that may be evaluated in this article, or claim that may be made by its manufacturer, is not guaranteed or endorsed by the publisher.

## References

[B1] Albecka A. Owen D. J. Ivanova L. Brun J. Liman R. Davies L. (2017). Dual function of the pUL7-pUL51 tegument protein complex in herpes simplex virus 1 infection. *J. Virol.* 91 e002196–e2116. 10.1128/JVI.02196-16 27852850 PMC5215335

[B2] Badr Y. Okada A. Abo-Sakaya R. Beshir E. Ohya K. Fukushi H. (2018). Equine herpesvirus type 1 ORF51 encoding UL11 as an essential gene for replication in cultured cells. *Arch. Virol.* 163 599–607. 10.1007/s00705-017-3650-4 29149435

[B3] Baines J. D. Roizman B. (1992). The UL11 gene of herpes simplex virus 1 encodes a function that facilitates nucleocapsid envelopment and egress from cells. *J. Virol.* 66 5168–5174. 10.1128/JVI.66.8.5168-5174.1992 1321297 PMC241400

[B4] Bender F. C. Whitbeck J. C. Ponce de Leon M. Lou H. Eisenberg R. J. Cohen G. H. (2003). Specific association of glycoprotein B with lipid rafts during herpes simplex virus entry. *J. Virol.* 77 9542–9552. 10.1128/jvi.77.17.9542-9552.2003 12915568 PMC187402

[B5] Briggs J. A. Wilk T. Fuller S. D. (2003). Do lipid rafts mediate virus assembly and pseudotyping? *J. Gen. Virol.* 84 757–768. 10.1099/vir.0.18779-0 12655075

[B6] Britt W. J. Jarvis M. Seo J. Y. Drummond D. Nelson J. (2004). Rapid genetic engineering of human cytomegalovirus by using a lambda phage linear recombination system: demonstration that pp28 (UL99) is essential for the production of infectious virus. *J. Virol.* 78 539–543. 10.1128/jvi.78.1.539-543.2004 14671136 PMC303398

[B7] Browman D. T. Hoegg M. B. Robbins S. M. (2007). The SPFH domain-containing proteins: more than lipid raft markers. *Trends Cell Biol.* 17 394–402. 10.1016/j.tcb.2007.06.005 17766116

[B8] Brown D. A. Rose J. K. (1992). Sorting of GPI-anchored proteins to glycolipid-enriched membrane subdomains during transport to the apical cell surface. *Cell* 68 533–544. 10.1016/0092-8674(92)90189-j 1531449

[B9] Chadha P. Han J. Starkey J. L. Wills J. W. (2012). Regulated interaction of tegument proteins UL16 and UL11 from herpes simplex virus. *J. Virol.* 86 11886–11898. 10.1128/JVI.01879-12 22915809 PMC3486286

[B10] Chazal N. Gerlier D. (2003). Virus entry, assembly, budding, and membrane rafts. *Mol. Biol. Rev.* 67 226–237. 10.1128/MMBR.67.2.226-237.2003 12794191 PMC156468

[B11] Cheng A. (2015). *Duck Plague.* Beijing: China Agriculture Press.

[B12] Claire M. M. Andrea L. K. Ekaterina E. H. (2020). Conserved outer tegument component UL11 from herpes simplex virus 1 is an intrinsically disordered, RNA-binding protein. *mBio* 11 e810–e820. 10.1128/mBio.00810-20 32371601 PMC7403781

[B13] Deng L. Wang M. Cheng A. Yang Q. Wu Y. Jia R. (2020). The pivotal roles of US3 protein in cell-to-cell spread and virion nuclear egress of duck plague virus. *Sci. Rep.* 10:7181. 10.1038/s41598-020-64190-2 32346128 PMC7189242

[B14] Dhama K. Kumar N. Saminathan M. Tiwari R. Karthik K. Kumar M. A. (2017). Duck virus enteritis (duck plague) - a comprehensive update. *Vet. Q.* 37 57–80. 10.1080/01652176.2017.1298885 28320263

[B15] Dietz A. N. Villinger C. Becker S. Frick M. von Einem J. (2017). A tyrosine-based trafficking motif of the tegument protein pul71 is crucial for human cytomegalovirus, secondary envelopment. *J. Virol.* 92 e00907–e917. 10.1128/JVI.00907-17 29046458 PMC5730796

[B16] Favoreel H. W. Mettenleiter T. C. Nauwynck H. J. (2004). Copatching and lipid raft association of different viral glycoproteins expressed on the surfaces of pseudorabies virus-infected cells. *J. Virol.* 78 5279–5287. 10.1128/jvi.78.10.5279-5287.2004 15113909 PMC400341

[B17] Fraser K. A. Rice S. A. (2007). Herpes simplex virus immediate-early protein ICP22 triggers loss of serine 2-phosphorylated RNA polymerase II. *J. Virol.* 81 5091–5101. 10.1128/JVI.00184-07 17344289 PMC1900222

[B18] Frasson I. Soldà P. Nadai M. Lago S. Richter S. N. (2021). Parallel G-quadruplexes recruit the HSV-1 transcription factor ICP4 to promote viral transcription in herpes virus-infected human cells. *Commun. Biol.* 4:510. 10.1038/s42003-021-02035-y 33931711 PMC8087788

[B19] Han J. Chadha P. Meckes D. G. Jr. Baird N. L. Wills J. W. (2011). Interaction and interdependent packaging of tegument protein UL11 and glycoprotein e of herpes simplex virus. *J. Virol.* 85 9437–9446. 10.1128/JVI.05207-11 21734040 PMC3165753

[B20] Han J. Chadha P. Starkey J. L. Wills J. W. (2012). Function of glycoprotein E of herpes simplex virus requires the coordinated assembly of three tegument proteins on its cytoplasmic tail. *Proc. Natl. Acad. Sci. U.S.A.* 109 19798–19803. 10.1073/pnas.1212900109 23150560 PMC3511771

[B21] He Q. Cheng A. Wang M. Xiang J. Zhu D. Zhou Y. (2012). Replication kinetics of duck enteritis virus UL16 gene in vitro. *Virol. J.* 21:281. 10.1186/1743-422X-9-281 23171438 PMC3560188

[B22] Johnson D. C. Baines J. D. (2011). Herpesviruses remodel host membranes for virus egress. *Nat. Rev. Microbiol.* 9 382–394. 10.1038/nrmicro2559 21494278

[B23] Kim I. J. Chouljenko V. N. Walker J. D. Kousoulas K. G. (2013). Herpes simplex virus 1 glycoprotein M and the membrane-associated protein UL11 are required for virus-induced cell fusion and efficient virus entry. *J. Virol.* 87 8029–8037. 10.1128/JVI.01181-13 23678175 PMC3700202

[B24] Kobty M. (2015). Herpes simplex virus: beyond the basics. *Neonatal. Netw.* 34 279–283. 10.1891/0730-0832.34.5.279 26802828

[B25] Kopp M. Granzow H. Fuchs W. Klupp B. Mettenleiter T. C. (2004). Simultaneous deletion of pseudorabies virus tegument protein UL11 and glycoprotein M severely impairs secondary envelopment. *J. Virol.* 78 3024–3034. 10.1128/jvi.78.6.3024-3034.2004 14990721 PMC353770

[B26] Kopp M. Granzow H. Fuchs W. Klupp B. G. Mundt E. Karger A. (2003). The pseudorabies virus UL11 protein is a virion component involved in secondary envelopment in the cytoplasm. *J. Virol.* 77 5339–5351. 10.1128/jvi.77.9.5339-5351.2003 12692236 PMC153988

[B27] Koshizuka T. Goshima F. Takakuwa H. Nozawa N. Daikoku T. Koiwai O. (2002). Identification and characterization of the UL56 gene product of herpes simplex virus type 2. *J. Virol.* 76 6718–6728. 10.1128/jvi.76.13.6718-6728.2002 12050385 PMC136277

[B28] Koshizuka T. Kawaguchi Y. Nozawa N. Mori I. Nishiyama Y. (2007). Herpes simplex virus protein UL11 but not UL51 is associated with lipid rafts. *Virus Genes* 35 571–575. 10.1007/s11262-007-0156-2 17694428

[B29] Labiuk S. L. Lobanov V. Lawman Z. Snider M. Babiuk L. A. van Drunen Littel-van den Hurk S. (2010). Bovine herpesvirus-1 US3 protein kinase: critical residues and involvement in the phosphorylation of VP22. *J. Gen. Virol.* 91 1117–1126. 10.1099/vir.0.016600-0 20016039

[B30] Lee G. E. Church G. A. Wilson D. W. (2003). A subpopulation of tegument protein vhs localizes to detergent-insoluble lipid rafts in herpes simplex virus-infected cells. *J. Virol.* 77 2038–2045. 10.1128/jvi.77.3.2038-2045.2003 12525638 PMC140989

[B31] Li Y. Wu Y. Wang M. Ma Y. Jia R. Chen S. (2020). Duplicate US1 genes of duck enteritis virus encode a non-essential immediate early protein localized to the nucleus. *Front. Cell. Infect. Microbiol.* 17:463. 10.3389/fcimb.2019.00463 32010642 PMC6979402

[B32] Loomis J. S. Bowzard J. B. Courtney R. J. Wills J. W. (2001). Intracellular trafficking of the UL11 tegument protein of herpes simplex virus type 1. *J Virol.* 75, 12209–12219. 11711612 10.1128/JVI.75.24.12209-12219.2001 PMC116118

[B33] Loomis J. S. Courtney R. J. Wills J. W. (2006). Packaging determinants in the UL11 tegument protein of herpes simplex virus type 1. *J Virol.* 80, 10534–10541. 10.1128/JVI.01172-06 16928743 PMC1641780

[B34] Lu Y. Liu D. X. Tam J. P. (2008). Lipid rafts are involved in SARS-CoV entry into Vero E6 cells. *Biochem. Biophys. Res. Commun.* 369 344–349. 10.1016/j.bbrc.2008.02.023 18279660 PMC7092920

[B35] Manes S. del Real G. Martinez A. C. (2003). Pathogens: raft hijackers. *Nat. Rev. Immunol.* 3 557–568. 12876558 10.1038/nri1129

[B36] Mettenleiter T. C. Klupp B. G. Granzow H. (2009). Herpesvirus assembly: an update. *Virus Res.* 143 222–234. 10.1016/j.virusres.2009.03.018 19651457

[B37] Oshima S. Daikoku T. Shibata S. Yamada H. Goshima F. Nishiyama Y. (1998). Characterization of the UL16 gene product of herpes simplex virus type 2. *Arch. Virol.* 143 863–880. 10.1007/s007050050338 9645194

[B38] Owen D. J. Crump C. M. Graham S. C. (2015). Tegument assembly and secondary envelopment of alphaherpesviruses. *Viruses* 7 5084–5114. 10.3390/v7092861 26393641 PMC4584305

[B39] Pereira E. A. daSilva L. L. (2016). HIV-1 nef: taking control of protein trafficking. *Traffic* 17 976–996. 10.1111/tra.12412 27161574

[B40] Po’ka N. Csabai Z. Pa’sti E. Tomba’cz D. Boldogkoi Z. (2017). Deletion of the us7 and us8 genes of pseudorabies virus exerts a differential effect on the expression of early and late viral genes. *Virus Genes* 53 603–612. 10.1007/s11262-017-1465-8 28477233

[B41] Qi X. Yang X. Cheng A. Wang M. Zhu D. Jia R. (2008). The pathogenesis of duck virus enteritis in experimentally infected ducks: a quantitative time- course study using taqman polymerase chain reaction. *Avian Pathol.* 37 307–310. 10.1080/03079450802043775 18568657

[B42] Qi X. Yang X. Cheng A. Wang M. Zhu D. Jia R. (2009). Intestinal mucosal immune response against virulent duck enteritis virus infection in ducklings. *Res. Vet. Sci.* 87 218–225. 10.1016/j.rvsc.2009.02.009 19303123

[B43] Ramachandran S. Davoli K. A. Yee M. B. Hendricks R. L. Kinchington P. R. (2010). Delaying the expression of herpes simplex virus type 1 glycoprotein B (gB) to a true late gene alters neurovirulence and inhibits the gB-CD8+ T-cell response in the trigeminal ganglion. *J. Virol.* 84 8811–8820. 10.1128/JVI.00496-10 20573821 PMC2919033

[B44] Rossman J. S. Lamb R. A. (2011). Influenza virus assembly and budding. *Virology* 411 229–236. 10.1016/j.virol.2010.12.003 21237476 PMC3086653

[B45] Schimmer C. Neubauer A. (2003). The equine herpesvirus 1 UL11 gene product localizes to the trans-golgi network and is involved in cell-to-cell spread. *Virology* 308 23–36. 10.1016/s0042-6822(02)00060-0 12706087

[B46] Simons K. Ikonen E. (1997). Functional rafts in cell membranes. *Nature* 387 569–572. 10.1038/42408 9177342

[B47] Takeda M. Leser G. P. Russell C. J. Lamb R. A. (2003). Influenza virus hemagglutinin concentrates in lipid raft microdomains for efficient viral fusion. *Proc. Natl. Acad. Sci. U.S.A.* 100 14610–14617. 10.1073/pnas.2235620100 14561897 PMC299746

[B48] Tang S. Patel A. Krause P. R. (2019). Hidden regulation of herpes simplex virus 1 pre-mRNA splicing and polyadenylation by virally encoded immediate early gene ICP27. *PLoS Pathog.* 15:e1007884. 10.1371/journal.ppat.1007884 31206552 PMC6597130

[B49] Watanabe M. Arii J. Takeshima K. Fukui A. Shimojima M. Kozuka-Hata H. (2021). Prohibitin-1 contributes to cell-to-cell transmission of herpes simplex virus 1 via the MAPK/ERK signaling pathway. *J. Virol.* 95 e1413–e1420. 10.1128/JVI.01413-20 33177205 PMC7925112

[B50] Yang L. Shen B. Wang M. Cheng A. Yang Q. Wu Y. (2021). The intracellular domain of duck plague virus glycoprotein E affects UL11 protein incorporation into viral particles. *Vet. Microbiol.* 257:109078. 10.1016/j.vetmic.2021.109078 33906107

[B51] Yang L. Wang M. Cheng A. Yang Q. Wu Y. Jia R. (2019). Innate immune evasion of alphaherpesvirus tegument proteins. *Front. Immunol.* 10:2196. 10.3389/fimmu.2019.02196 31572398 PMC6753173

[B52] Yang L. Wang M. Zeng C. Shi Y. Cheng A. Liu M. (2020). Duck enteritis virus UL21 is a late gene encoding a protein that interacts with pUL16. *BMC Vet. Res.* 16:8. 10.1186/s12917-019-2228-7 31915010 PMC6950997

[B53] Yokoyama H. Matsui I. (2020). The lipid raft markers stomatin, prohibitin, flotillin, and HflK/C (SPFH)-domain proteins form an operon with NfeD proteins and function with apolar polyisoprenoid lipids. *Crit. Rev. Microbiol.* 46 38–48. 10.1080/1040841X.2020.1716682 31983249

[B54] You Y. Cheng A. Wang M. Jia R. Sun K. Yang Q. (2017). The suppression of apoptosis by a-herpesvirus. *Cell Death Dis.* 8:e2749. 28406478 10.1038/cddis.2017.139 PMC5477576

[B55] Yuan G. Cheng A. Wang M. Han X. Zhou Y. Liu F. (2007). Preliminary study on duck enteritis virus-induced lymphocyte apoptosis in vivo. *Avian Dis.* 51 546–549. 10.1637/0005-2086(2007)51[546:PSODEV]2.0.CO;2 17626481

[B56] Zeev-Ben-Mordehai T. Hagen C. Grünewald K. (2014). A cool hybrid approach to the herpesvirus “life” cycle. *Curr. Opin. Virol.* 5 42–49. 10.1016/j.coviro.2014.01.008 24553093 PMC4031633

