# Supplementary material for: UL11 Protein Is a Key Participant of the Duck Plague Virus in Its Life Cycle
Source: Front Microbiol. 2022 Jan 4;12:792361. doi: 10.3389/fmicb.2021.792361 (PMC8764364; doi:10.3389/fmicb.2021.792361)

Original image data used in the article

Figure 1A

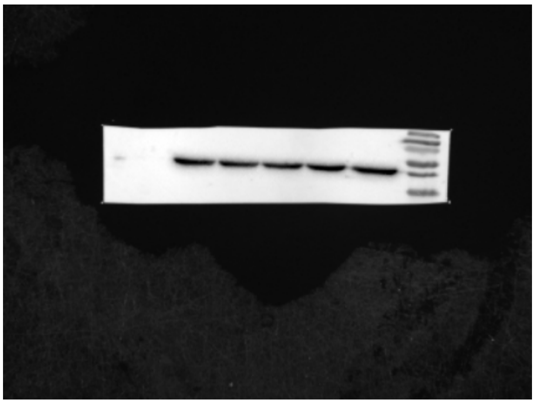

β-actin

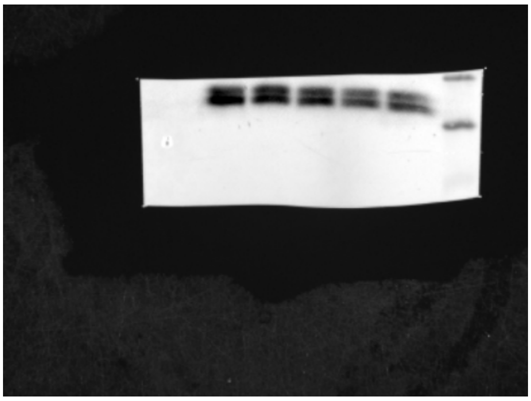

UL11

Figure 2E

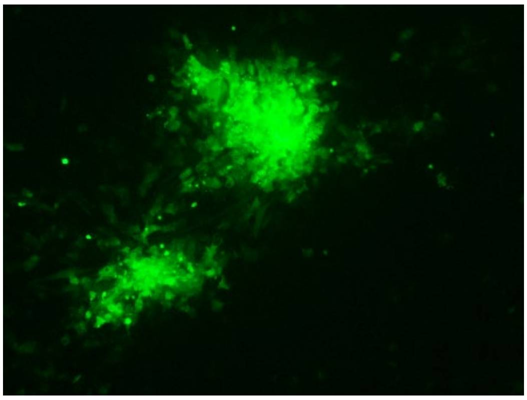

shRNAneo

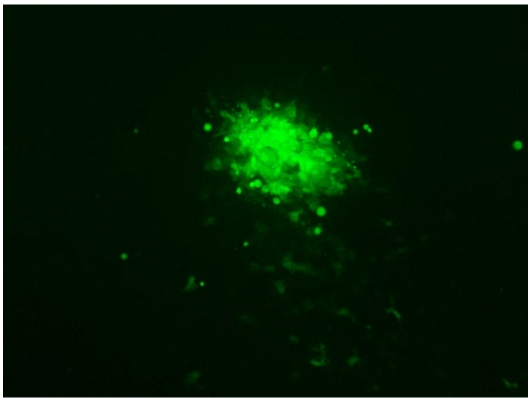

shRNA126

Figure 3

shRNA126：

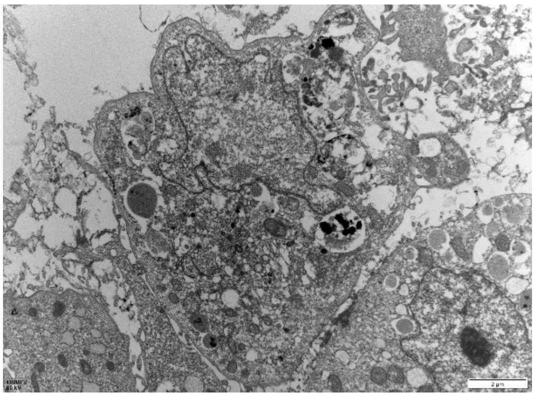

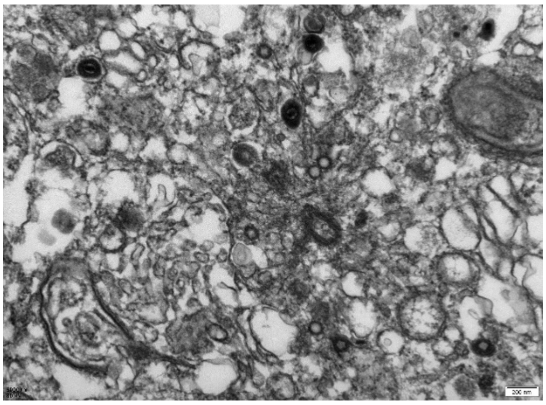

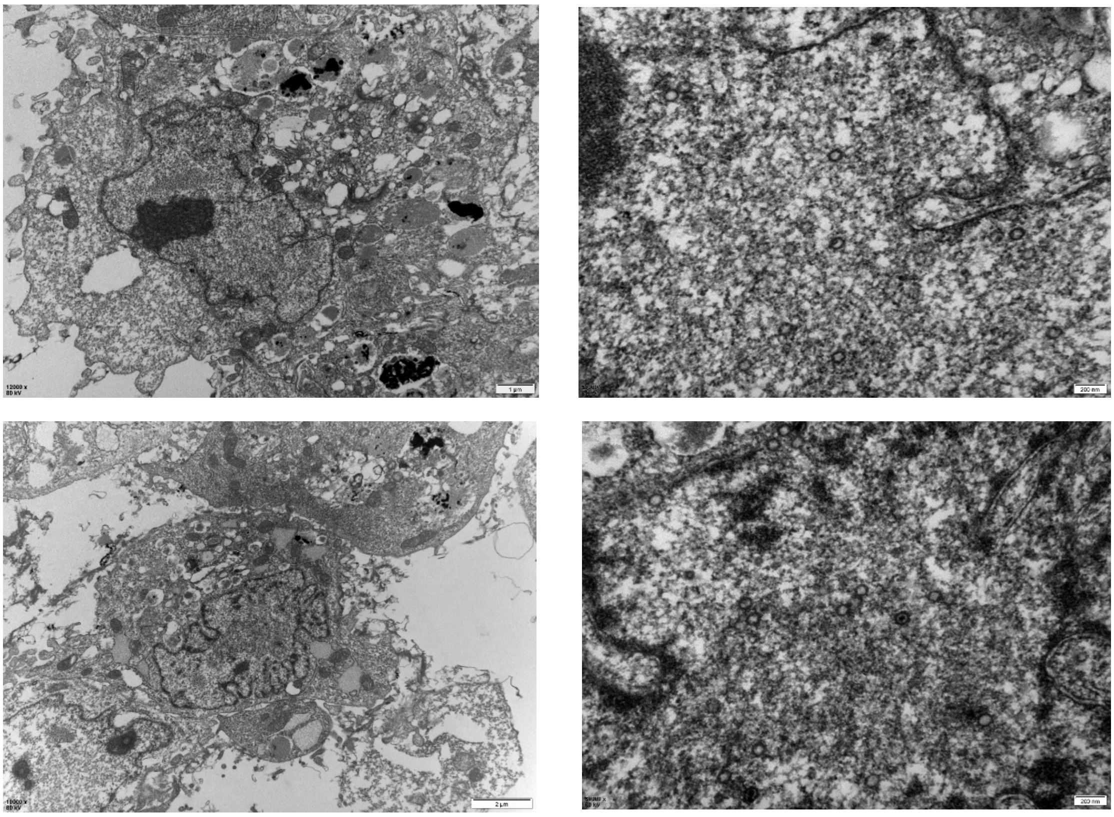

shRNAneo：

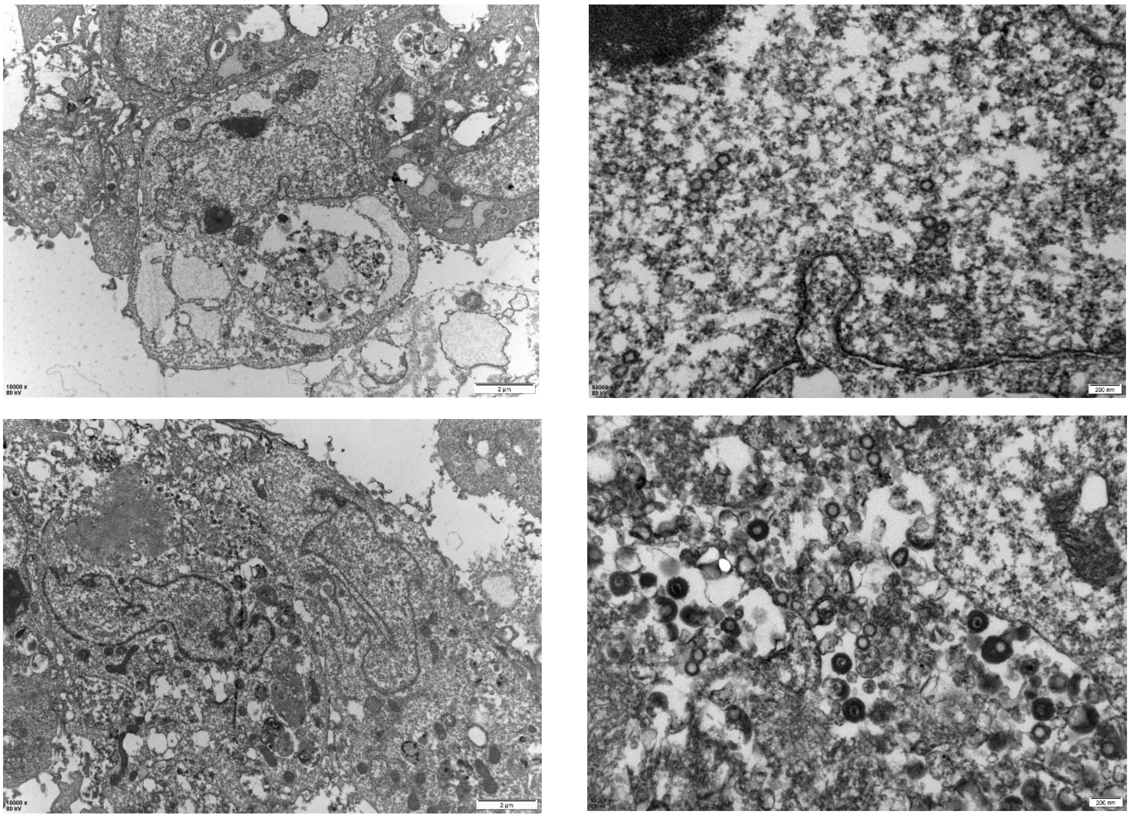

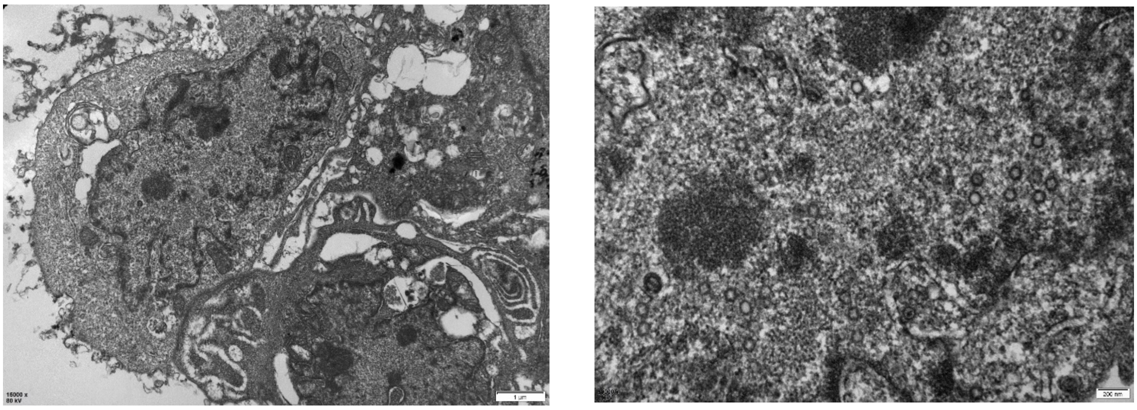

Figure 5A

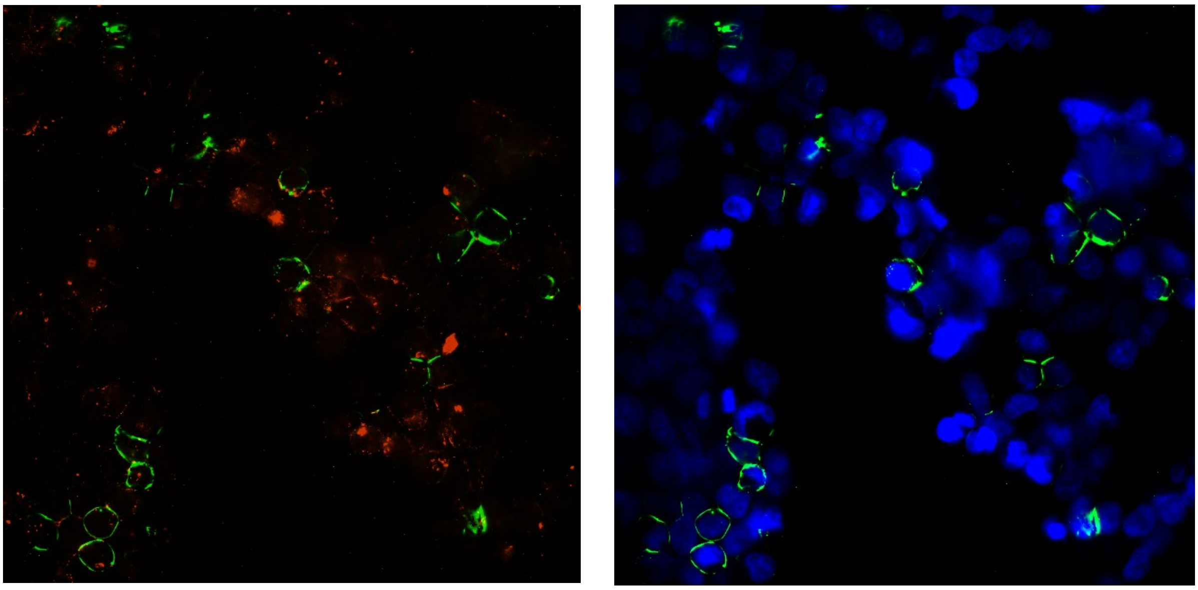

Figure 5C

UL11

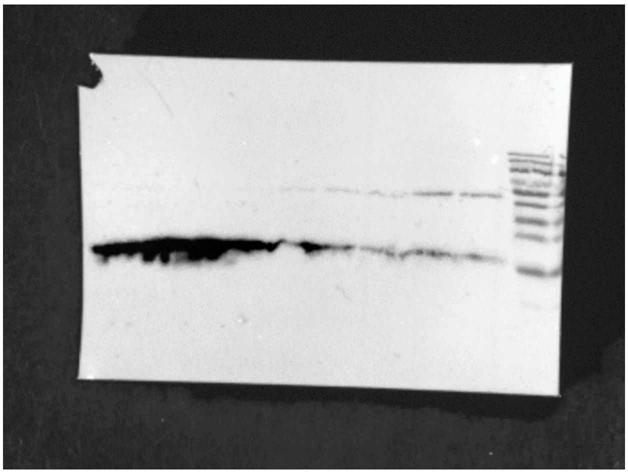

PHB

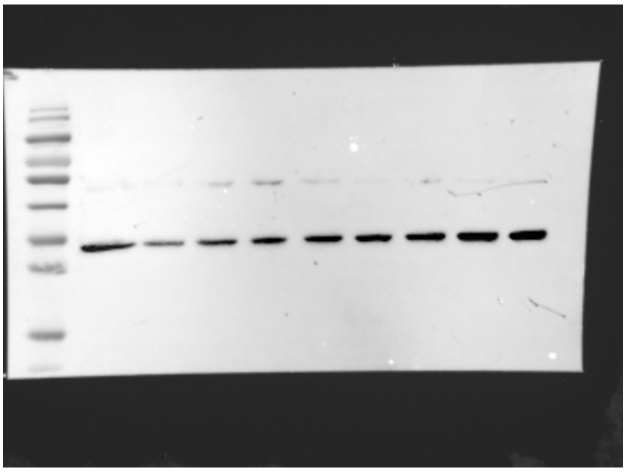

FLOT1

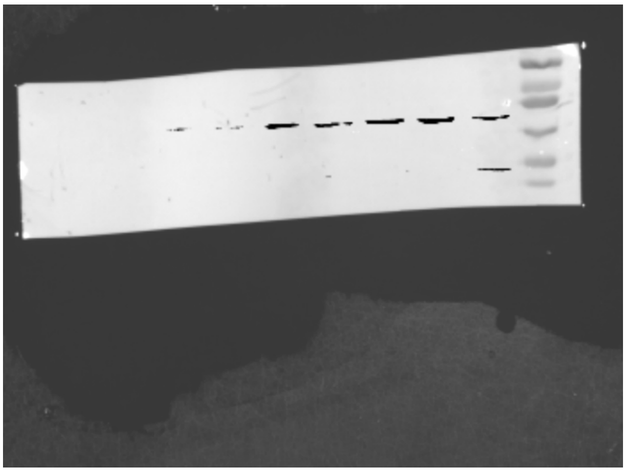

Figure 5D

UL11G2A

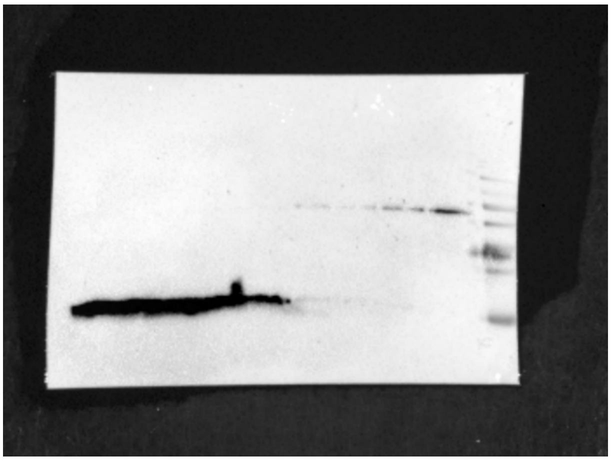

PHB

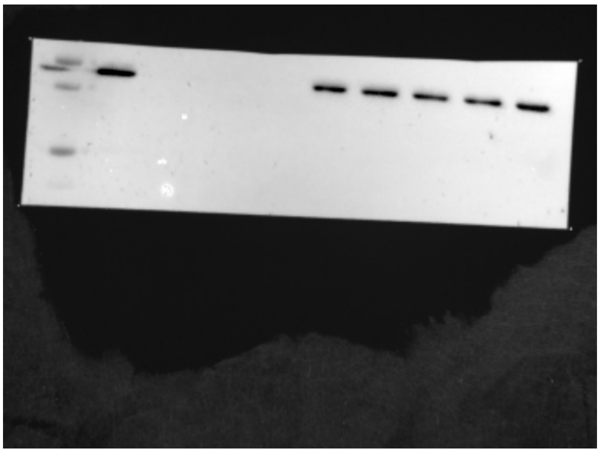

FLOT1

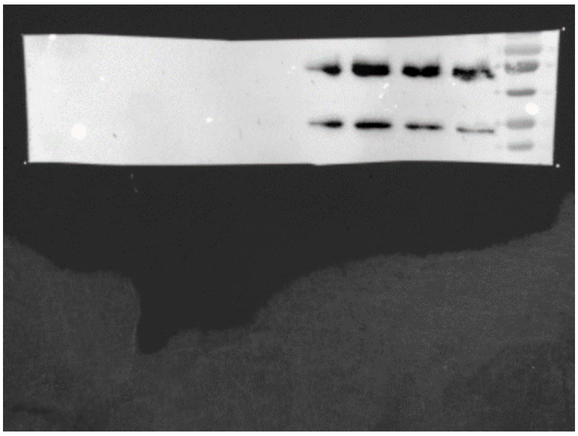

Figure 6C

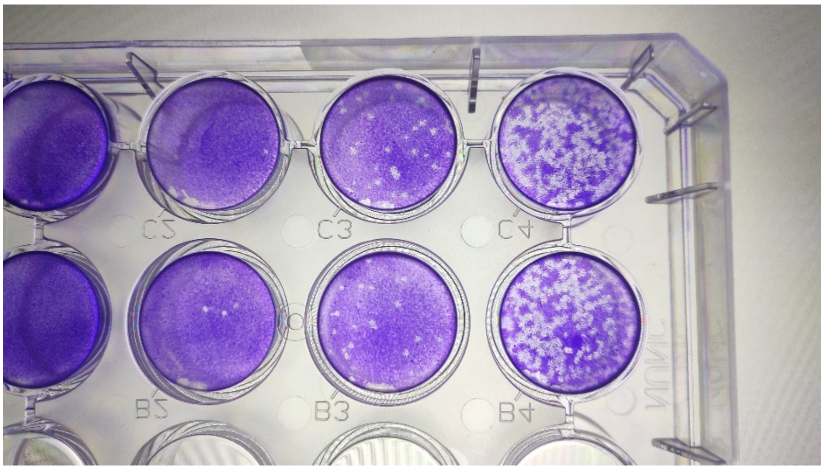

Supplement: Supplementary file 1 [file Data_Sheet_1.pdf]
