# Supplementary material for: UL11 Protein Is a Key Participant of the Duck Plague Virus in Its Life Cycle
Source: Front Microbiol. 2022 Jan 4;12:792361. doi: 10.3389/fmicb.2021.792361 (PMC8764364; doi:10.3389/fmicb.2021.792361)
Supplement: Supplementary file 3 [file Data_Sheet_3.docx]

Supplementary Material

##
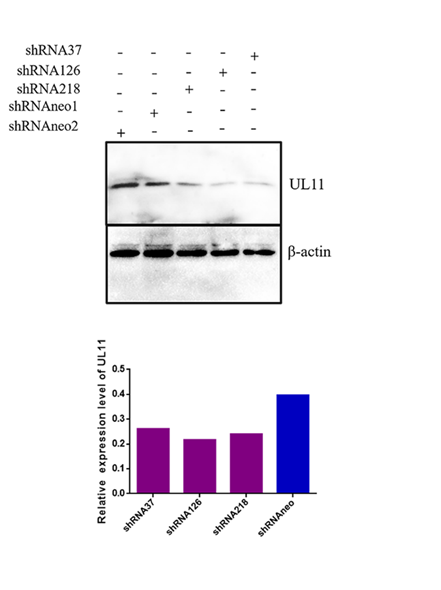
Supplementary Figures

**Supplementary Figure 1.** Selection and validation of UL11 shRNA. DEF cells were firstly transfected with shRNA37, shRNA126, shRNA218, shRNAneo, and then incubated with 1MOI DPV for 24 h, cells were collected to do western blot analysis. shRNAneo1 and shRNAneo2 are two negative control.


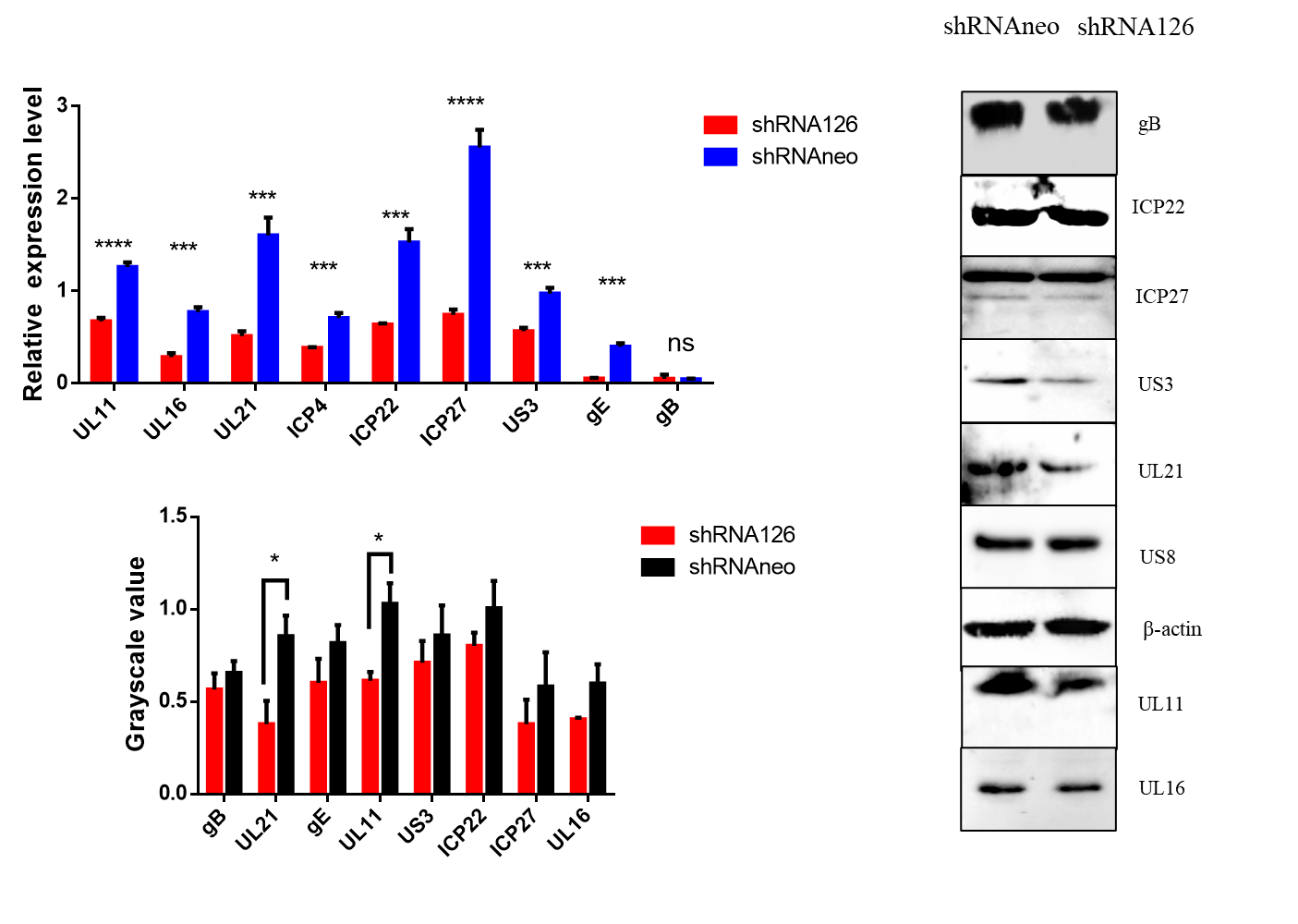


**Supplementary Figure 2.** Protein expression level of UL11, UL16, UL21, gE, ICP22, ICP27, US3 and gB when interfere the UL11 gene.
